# Increasing childhood vaccination coverage of the refugee and migrant population in Greece through the European programme PHILOS, April 2017 to April 2018

**DOI:** 10.2807/1560-7917.ES.2019.24.27.1800326

**Published:** 2019-07-04

**Authors:** Kassiani Mellou, Chrysovalantis Silvestros, Eirini Saranti-Papasaranti, Athanasios Koustenis, Ioanna D. Pavlopoulou, Theano Georgakopoulou, Chryssoula Botsi, Agapios Terzidis

**Affiliations:** 1 Hellenic Centre for Disease Control and Prevention, Athens, Greece; 2 National and Kapodistrian University of Athens, Faculty of Nursing, ‘P & A Kyriakou’ Children’s Hospital, Athens, Greece

**Keywords:** childhood vaccination, refugees, accommodation camps, vaccination coverage, immunisation, Greece, measles-mumps-rubella (MMR) vaccine, MMR, public health policy, vaccines

## Abstract

After the 2016 Balkan route border closures, vaccination of refugee children in Greece was mainly performed by non-governmental organisations. Activities varied between camps, resulting in heterogeneity of vaccination coverage (VC). In April 2017, the European programme ‘PHILOS - Emergency health response to refugee crisis’ took over vaccination coordination. Interventions were planned for the first time for refugee children in the community and unaccompanied minors at safe zones. From April 2017–April 2018, 57,615 vaccinations were performed against measles-mumps-rubella (MMR) (21,031), diphtheria-tetanus-pertussis (7,341), poliomyelitis (7,652), pneumococcal disease (5,938), *Haemophilus influenzae* type b (7,179) and hepatitis B (8,474). In April 2018, the vaccination status of children at camps (reception and identification centres and community facilities such as hostels/hotels were excluded) was recorded and VC for each disease, stratified by dose, nationality and camp size, was calculated. More than 80% of the children received the first MMR dose, with VC dropping to 45% for the second dose. For all other vaccines, VC was < 50% for the first dose in children aged 0–4 years and < 25% for the second dose. Despite challenges, PHILOS improved planning and monitoring of vaccination activities; however, further efforts towards improving VC in refugee children are needed.

## Background

In 2015, during the refugee crisis, over one million refugees and migrants journeyed across the Mediterranean Sea to Europe; in 2016 and 2017, a further 535,054 people arrived in Europe through the same route [1].

Greece is one of the major entry points of Europe. After the Balkan route border closures in 2016, thousands of refugees who entered Greece were not able to proceed towards their intended country of refuge and had to remain in Greece [2]. In 2016, 2017 and 2018 (January to April), 173,450, 29,718 and 8,362 people, respectively, arrived at Greece’s north-eastern Aegean Islands, with children < 15 years of age accounting for 38% of the total population [1]. In 2016, the Hellenic Immunisation Advisory Committee recommended vaccination against the following priority diseases for these newly arrived children: measles, mumps and rubella for children 1–14 years old and diphtheria, tetanus, pertussis, poliomyelitis, pneumococcal disease, *Haemophilus influenzae* type b and hepatitis B for children 2 months–4 years old [3].

Until March 2017, vaccination of refugee children was mainly performed by non-governmental organisations (NGOs) under the coordination of the Greek Ministry of Health (MoH). Vaccination activities varied by camp and not all camps were consistently covered by an NGO [4]. Moreover, the vaccination needs of the population living in the community (hostels, hotels, apartments, etc.) or ‘safe zones’ for unaccompanied minors, were not addressed in this phase. This lack of harmonisation of vaccination practices and the resulting heterogeneity of vaccination coverage (VC) was a public health concern.

Therefore, in April 2017 it was decided that the programme ‘PHILOS – Emergency health response to refugee crisis’ would take over the coordination of vaccination of refugee children in Greece. PHILOS was a programme of the Greek MoH, implemented by the Hellenic Centre for Disease Control and Prevention (HCDCP) and funded by the Asylum, Migration and Integration Fund (AMIF) of the European Union’s Directorate-General for Migration and Home Affairs. All activities were implemented under the supervision of the General Directorate of Public Health of the MoH.

Here we present the activities of the PHILOS programme from April 2017–April 2018 and the results of the concluding assessment of VC in April 2018 of refugee children in Greece, by disease, as well as the challenges faced in the programme’s implementation.

In this manuscript, we refer to refugees, asylum seekers and newly arrived migrants as refugees.

## Setting

In recent years, refugees have entered Greece mainly through the north-eastern Aegean Islands. They are then placed at RICs (each island has one RIC) and are offered medical assessment and medical care, if needed. Depending on their asylum or vulnerability status, most people are then transferred to accommodation camps on the mainland, where they stay for a more extended period until they are finally hosted at hostels, hotels, apartments or other community facilities under the initiatives of the United Nations High Commissioner for Refugees (UNHCR) and other European Civil Protection and Humanitarian Aid Operations (ECHO) partners.

Unaccompanied children are initially placed at specially designed areas inside camps and RICs called ‘safe zones’, where they stay for a short period of time before they are transferred to community facilities.

## Vaccination activities at camps and reception and identification centres

The PHILOS programme set up standard operating procedures (SOPs) regarding vaccination practices at camps and RICs. Each NGO had to inform the PHILOS team about planned vaccination activities and had to request permission from the MoH to proceed with vaccinations. From April 2017 to April 2018, Red Cross, Praksis, Doctors Without Borders (MSF) and Doctors of the World (MdM) supported vaccination at camps, alongside the ‘Health for All’ programme of the University of Athens [5]. Camps not covered by an NGO were assigned to PHILOS mobile unit teams and all interventions were organised with the cooperation of the Ministry of Migration Policy.

Before each vaccination intervention, assessment of vaccination needs was required. To overcome the challenges posed by the mobility of the population and the heterogeneity of previously implemented interventions, PHILOS personnel visited families door-to-door and actively recorded the vaccination status of all children 0–14 years of age using a standardised form, within 2 weeks of each vaccination intervention. The resulting data were deposited in a specially designed database and sent to the HCDCP, in accordance with Greece’s legal framework for sensitive data protection.

All refugees were informed that they could opt-out of sharing their vaccination history or having their children vaccinated; however, written informed consent was not required given the practical limitations in the field.

The recorded information included: (i) a list of all the children 0–14 years of age hosted at each camp and their demographic characteristics (age, sex, nationality), (ii) each child’s exact place of residence (container/isobox) inside the camp or other information needed to locate them in future for vaccination, (iii) whether a child already had the World Health Organization (WHO) booklet or other type of vaccine documentation, (iv) any vaccines that a child had already received and (v) if a child had never been vaccinated or whose vaccination status was unknown. Collected data were used to establish the needs of refugee children at each individual camp, as well as at all of the camps as a whole, for better coordination of interventions.

While conducting the door-to-door household survey, personnel also informed the refugees of the planned vaccinations, their importance and how they would benefit the children. Whenever possible, the surveys were conducted with the support of cultural mediators to address possible queries from the population. Personnel also advised parents/guardians to keep their children’s vaccine records for future reference and informed them of the importance of these documents for the children’s registration at schools. Written information was also provided in English, Farsi, Urdu and Arabic.

A medical and a managerial coordinator were appointed for each intervention, in accordance with Greek law, which states that vaccination can only be performed in the presence of a medical doctor, preferably a paediatrician.

After the end of each intervention, vaccination teams reported the number of vaccinations performed to the HCDCP. Challenges encountered during interventions were also reported. All data were recorded in a common database.

Interventions were also organised at safe zones to accommodate unaccompanied minors, after permission was granted by the Ministry of Migration Policy to organise such an intervention and to contact unaccompanied minors. The Ministry of Migration Policy informed the district attorneys that act as the legal guardians of unaccompanied minors in Greece about the interventions and their scope.

## Planning vaccination activities for refugee children living in the community

As refugees were increasingly housed in hostels, hotels and apartments under the UNHCR initiatives, it was difficult to capture VC and needs. Therefore, a meeting with the UNHCR and their associated partner NGOs was organised to assess the VC of the refugee children living in the community and identify opportunities for better vaccination coordination. The NGO representatives at the meeting indicated that the children’s VC was low and that the majority of children in their premises were unvaccinated for most of the diseases included in the Greek National Childhood Immunisation Programme (NCIP). It was agreed that the HCDCP and the NGOs’ representatives would collaborate closely to address this.

For this reason, each of Greece’s seven health regions designated at least two community healthcare centres as vaccination centres that would cover the needs of the refugee child population living in the community. Vaccines and vaccination booklets were sent to the vaccination centres, and NGOs booked the appointments and trained the refugees on how to access the healthcare system.

## Vaccination campaigns

Vaccinations at camps and RICs were delivered through mass vaccination campaigns. The number of campaigns held at each camp depended on the size of the hosted population, the amount of new arrivals and the site’s resources. At minimum, camps were to perform a vaccination campaign at least once every 2 months.

Overall, from April 2017–April 2018, a total of 57,615 vaccinations were performed by NGOs and PHILOS in 15 and 10 camps, respectively: 21,031 against measles-mumps-rubella (MMR), 7,341 against diphtheria-tetanus-pertussis (DTP), 7,652 against poliomyelitis, 5,938 against pneumococcal disease, 7,179 against *Haemophilus influenzae* type b and 8,474 against hepatitis B.

Of the conducted vaccinations, 24,241 (42.1%) were performed at camps on the mainland, 17,649 (30.6%) at RICs, 14,941 (25.9%) in the community and 784 (1.4%) in safe zones.

## Assessment of vaccination coverage at mainland camps, April 2018

An assessment to estimate the VC of refugee children living in camps on the mainland took place in April 2018. PHILOS personnel visited the mainland camps over 2 weeks and went door-to-door to identify children aged 0–14 years and their vaccination status, based on WHO booklets or other documents they had been provided after their arrival to Greece. Parents’ statements regarding prior vaccinations were not taken into account.

The proportion of children 1–14 years of age that had been vaccinated against MMR and the proportion of children 0–4 years of age that had been vaccinated against diphtheria-tetanus-pertussis, poliomyelitis, pneumococcal disease, *Haemophilus influenzae* type b and hepatitis B—by dose, sex and nationality—were calculated. The association between the VC for at least one dose of each vaccine and the size of the camp (1–99 hosted children, ≥ 100 hosted children) was assessed.

In total, 3,786 children were recorded at the 25 camps on the mainland; 3,501 were 1–14 years of age and 1,509 were 0–4 years of age. Of these, 78.9% had the WHO booklet (2,572/3,261 for whom this information was available). Of the 3,722 children for whom the information was available, 2,002 were male (53.8%) and the mean age was 6 years (standard deviation (SD) ± 4.16). Overall, 22 different nationalities were recorded. Demographic data of children at the time of the survey are summarised in Table 1.

**Table 1:** Demographic characteristics of refugee children at 25 mainland camps, Greece, April 2018 (n = 3,786)

Characteristics		Children	
		n	%
Sex	Male	2,002	52.9
	Female	1,720	45.4
	Unknown	64	1.7
Age group (years)	< 1	285	7.5
	1–4	1,224	32.3
	5–14	2,277	60.2
Nationality	Syria	1,591	42.0
	Iraq	1,066	28.2
	Afghanistan	749	19.8
	Other^a^	375	9.9
	Unknown	5	0.1

^a^ There were 19 different nationalities recorded for the 375 children in the category of ‘Other’.

VC by disease is presented in Table 2. More than 80% of the children 1–14 years of age (2,843/3,501) had been vaccinated with the first dose of the MMR vaccine; however, for the second dose the coverage dropped to 45% (1,575/3,501). Coverage among children 0–4 years of age was < 50% for the first dose of all the other vaccines and below 25% for the second dose.

**Table 2:** Vaccination coverage^a^ of refugee children at 25 mainland camps, Greece, April 2018

Disease	Vaccine doses	Number of vaccinated children/total number of children recorded	%	
MMR	First	2,843/3,501		81.2
	Second	1,575/3,501		45.0
DTP	First	699/1,509		46.3
	Second	369/1,509		24.5
Poliomyelitis	First	704/1,509		46.7
	Second	370/1,509		24.5
Pneumococcal disease	First	750/1,509		49.7
	Second	267/1,509		17.7
*Haemophilus influenzae* type b	First	697/1,509		46.2
	Second	370/1,509		24.5
Hepatitis B	First	733/1,509		48.6
	Second	372/1,509		24.7

DTP: Diphtheria-tetanus-pertussis; MMR: measles-mumps-rubella.

^a^ The National Immunisation Advisory Committee in Greece recommends vaccination against MMR for children 1–14 years of age (n = 3,501) and against diphtheria-tetanus-pertussis, poliomyelitis, pneumococcal disease, *Haemophilus influenzae* type b and hepatitis B for children 0–4 years of age (n = 1,509). Vaccination campaigns followed the aforementioned recommendations; thus, vaccination coverage for each disease is presented for the respective age group.

The number of vaccinated children by sex and nationality is presented in Table 3. Sex did not have a statistically significant association with VC. The proportion of children vaccinated at each camp had a statistically significant association with nationality. Children from Afghanistan had higher VC compared with Syrian and Iraqi children. Larger camps had higher VC for at least one dose of MMR vaccine (p = 0.016) (Table 4). This difference appeared consistently for all other vaccines when looking at the point values, albeit without statistical significance.

**Table 3:** Number of vaccinated^a^ refugee children at 25 mainland camps, by sex and nationality, Greece, April 2018 (n=3,786)

Disease	Vaccine doses^b^	Sex^c^					Nationality^d^						
		Male		Female		p value^e^	Syria		Iraq		Afghanistan		p value^e^
		n	%	n	%		n	%	n	%	n	%	
MMR	First	1,528	82.0	1,299	82.1	0.975	1,166	80.1	811	79.8	608	88.0	0.001
	Second	828	44.4	746	47.1	0.115	586	40.2	418	41.1	445	64.4	< 0.001
DTP	First	363	46.8	334	47.8	0.698	296	40.7	144	40.0	192	69.1	< 0.001
	Second	192	24.8	177	25.4	0.796	142	19.5	66	18.3	128	46.0	< 0.001
Poliomyelitis	First	366	47.2	336	48.1	0.726	300	41.3	145	40.3	192	69.1	< 0.001
	Second	193	24.9	177	25.4	0.841	143	19.7	66	18.3	128	46.0	< 0.001
Pneumococcal disease	First	394	50.8	355	50.9	0.994	336	46.2	153	42.5	194	69.8	< 0.001
	Second	146	18.8	121	17.3	0.455	98	13.5	39	10.8	111	39.9	< 0.001
*Haemophilus influenzae* type b	First	363	46.8	332	47.6	0.781	294	40.4	144	40.0	192	69.1	< 0.001
	Second	193	24.9	177	25.4	0.841	143	19.7	66	18.3	128	46.0	< 0.001
Hepatitis B	First	381	49.2	350	50.1	0.707	316	43.5	151	41.9	194	69.8	< 0.001
	Second	193	24.9	179	25.6	0.744	145	19.9	66	18.3	128	46.0	< 0.001

DTP: Diphtheria-tetanus-pertussis; MMR: measles-mumps-rubella.

^a^ For the calculation of the vaccination coverage for MMR that refers to children 1–14 years of age (n = 3,501) the respective denominators were used: 1,863 of the children were male, 1,583 female, 1,456 were from Syria, 1,016 from Iraq and 691 from Afghanistan. Similarly, for the calculation of vaccination coverage for diphtheria-tetanus-pertussis, poliomyelitis, pneumococcal disease, *Haemophilus influenzae* type b and hepatitis B that refers to children 0–4 years of age (n = 1,509), the following denominators were used: 775 children were male, 698 females 727 were from Syria, 360 from Iraq and 278 from Afghanistan.

^b^ Coverage for more doses of the aforementioned vaccines (when applicable) was also recorded; however, the data was not presented here due to the low numbers of vaccinated children.

^c^ Information regarding sex was missing for 64 records.

^d^ Information regarding nationality was missing for five records. The other 375 children of 19 different nationalities are not included in the table.

^e^ Pearson’s chi-squared test p value.

**Table 4:** Vaccination coverage^a^ of refugee children at 25 mainland camps, Greece, April 2018 (n=3,786)

Disease	Vaccine doses	Camp size (1–99 children)	Camp size (≥ 100 children)	p value^b^
		Mean (± SD)	Mean (± SD)	
MMR	No dose	39.9 (± 32.28)	16.9 (± 11.43)	0.016
	At least one dose	60.1 (± 32.28)	83.06 (± 11.43)	
DTP^c^	No dose	69.2 (± 13.15)	56.2 (± 19.85)	0.094
	At least one dose	30.8 (± 13.15)	43.8 (± 19.85)	
Poliomyelitis	No dose	69.2 (± 13.15)	56.0 (± 20.04)	0.090
	At least one dose	30.8 (± 13.15)	44.0 (± 20.04)	
Pneumococcal disease	No dose	56.1 (± 29.40)	56.4 (± 19.67)	0.977
	At least one dose	43.9 (± 29.40)	43.6 (± 19.67)	
*Haemophilus influenzae* type b	No dose	69.7 (± 13.00)	56.2 (± 19.84)	0.081
	At least one dose	30.3 (± 13.00)	43.8 (± 19.84)	
Hepatitis B	No dose	68.5 (± 13.66)	52.8 (± 22.75)	0.073
	At least one dose	31.5 (± 13.66)	47.2 (± 22.75)	

DTP: Diphtheria-tetanus-pertussis; MMR: measles-mumps-rubella; SD: standard deviation.

^a^ Vaccination coverage against MMR for children 1–14 years of age (n = 3,501) and vaccination coverage against diphtheria-tetanus-pertussis, poliomyelitis, pneumococcal disease, *Haemophilus influenzae* type b and hepatitis B for children 0–4 years of age (n = 1,509), by camp size (1–99 children and ≥ 100).

^b^ Student's t-test p-value.

## Lessons learnt and challenges

Despite the positive outcome of PHILOS, a number of challenges were encountered while the programme was active.

### Availability of cultural mediators

PHILOS and NGO personnel had to deal with an insufficient number of cultural mediators on site, a well-documented challenge in such settings [6,7]. Additionally, the camps’ populations were constantly changing, with people moving from one camp to another or to community shelters. Eight camps closed in 2017 and two new ones opened, making vaccination status follow-up and planning of vaccination campaigns difficult.

To address this, vaccination campaigns were designed to be flexible and in several cases campaigns were postponed to assure the presence of cultural mediators. In other cases, campaigns had to be prolonged in order to cover newcomers or unexpected needs. All available human resources that could support mediation were utilised. In addition to NGO and PHILOS personnel, volunteers from the community and in some cases members of the camps’ populations also supported vaccination efforts.

### Limitations of recording VC

The results of the survey in April 2018 were useful, as constant monitoring of VC in the camps via a specially designed vaccination registry was not possible. However, the estimated coverage only accounts for the 3,786 children recorded as living in the camps in April 2018 and cannot be extrapolated to the population living in RICs or in the community, or to those who occupied the camps in the following months. RICs were not included in the study because the increased workload for the medical staff and the continuous arrival of refugees made door-to-door surveying impossible.

### Insufficient vaccination coverage remained for a number of diseases

As at April 2018, VC was high for the first dose of the vaccine against MMR, but was far from optimal for the second dose, as well as for the first and second dose against pneumococcal disease, hepatitis B, poliomyelitis, diphtheria-tetanus-pertussis and *Haemophilus influenzae* type b. Therefore, mass vaccination campaigns were still needed. During the months following the assessment, new campaigns were designed, giving priority to the camps with the lowest VC.

The use of a combination vaccine that protects against diphtheria, tetanus, pertussis, poliomyelitis, hepatitis B and infection with *Haemophilus influenzae* type B bacteria in most of the vaccination campaigns explains the similar VC for all six diseases.

### Differing vaccination coverage by nationalities and camp size

The difference in VC by nationality may be attributed to the quicker turnover of the Syrian population at camps, as they had more straightforward access to the asylum processes. Syrian children represented in the study may have missed prior vaccination campaigns if they had recently entered the camps. However, this is just a hypothesis, as the dates of arrival at the camps were not recorded.

The higher vaccination coverage at larger camps may be explained by the fact that vaccination campaigns might have been more organised, frequent and effective compared with those at smaller ones.

### Increased refugee childhood population in the community

Vaccination activities for refugee children living in the community at hotels, hostels or private houses were designed for the first time; however, the estimated number of unvaccinated children in the community was beyond the purposes of this study. By the end of April 2018, the number of refugees living in the community had increased to more than 20,000, 48% of whom were children [8]. As the number of refugee children in the community steadily became greater than the number of children hosted at the mainland camps, priority had to be given to this population. Therefore, vaccination interventions began to be implemented in the community, not only at the camps, as was done previously. During this period, the PHILOS programme focused on covering vaccination gaps, with the support of health regions as previously described, and guiding families living outside camps to acquire national health security numbers that would allow for vaccinations based on the NCIP, free of charge.

### Conclusion

Vaccination of refugee children is a priority for host countries; however, several studies have documented that refugees have low immunisation rates and encounter essential barriers in accessing routine healthcare services.

In 2017, the PHILOS programme took over the coordination of refugee children’s vaccinations in Greece. PHILOS set standard operating procedures for identifying vaccination needs at camps and RICs, performing information campaigns with the support of cultural mediators and promoting the use of a singular booklet for documenting vaccination history for all children. PHILOS’ activities showed that the implementation of a coordinated approach to vaccinations in such a complex situation and setting is feasible and provided a useful experience of cooperation between the HCDCP, MoH, regional public health authorities, international organisations, NGOs and other stakeholders in organising vaccination campaigns. Overall, the programme demonstrated progress in the coordination of vaccinations for refugee children in Greece. Vaccination of all refugee children upon arrival and enhancement of continued access to healthcare should be future public health priorities.
